# Nucleoporin 160 (NUP160) inhibition alleviates diabetic nephropathy by activating autophagy

**DOI:** 10.1080/21655979.2021.1968777

**Published:** 2021-09-17

**Authors:** Jiayong Xie, Ying Yuan, Gang Yao, Zhi Chen, Wenjuan Yu, Qiang Zhu

**Affiliations:** a Department of Nephrology, Xinghua People’s Hospital, Taizhou Jiangsu, China; b Department of Nephrology, Second Affiliated Hospital of Nanjing Medical University, Nanjing Jiangsu, China; c Department of Laboratory, Xinghua People’s Hospital, Taizhou Jiangsu, China

**Keywords:** NUP160, autophagy, diabetic nephropathy, inflammatory response

## Abstract

Diabetic nephropathy (DN) is the leading cause of end-stage renal disease worldwide. Autophagy was reported to be related to the pathogenesis of DN. This research investigated the function of the Nucleoporin 160 (Nup160) gene in regulating autophagy in DN. A mouse model of DN was established through an intraperitoneal injection of streptozotocin (STZ). Normal rat kidney tubular epithelial cells (NRK-52E) were treated with high glucose to induce DN in vitro. Real-time quantitative polymerase chain reaction (RT-qPCR), western blot, immunofluorescence assays were conducted to measure the expression of NUP160, autophagy-associated proteins, and inflammatory cytokines in vitro and in vivo. Pathological changes of kidney and liver tissues were analyzed using hematoxylin and eosin (H&E), Masson and periodic acid-silver (PAS) staining. The body weight, blood glucose, renal and lipid profiles of DN mice were examined. In this study, DN mice showed serious pathological injury. NUP160 expression was upregulated, autophagy was inhibited, and inflammatory response was increased in DN mice. Depletion of NUP160 restored autophagy and inhibited inflammation and fibrosis in high glucose (HG)-treated NRK-52E cells and STZ-induced DN mice by downregulating the expression of p62 and Collagen IV (Col-Ⅳ), increasing the ratio of LC3II/LC3I, and inactivating nuclear factor (NF)-κB signaling. Moreover, NUP160 knockdown could ameliorate pathological damage and glucose tolerance in DN mice. Overall, this study is the first to demonstrate the key role of NUP160 silencing in promoting autophagy against diabetic injury in DN.

## Introduction

In recent years, the prevalence of diabetes has been on the rise, and it is the leading cause of human disease and premature death worldwide [1]. Diabetic nephropathy (DN) is a complication of diabetes that induces the development of end-stage renal disease [2,3]. Approximately 30% of diabetic individuals suffering from DN develop end-stage renal failure [4,5]. DN is accompanied by elevation of urinary albumin and blood pressure and loss of glomerular filtration [6,7]. Studies show that the disturbance of internal environment and metabolism caused by hyperglycemia is the risk factor of DN [8]. Renal tubulointerstitial fibrosis is common in DN, which is characterized by mesangium thickening, tubular atrophy and abnormal accumulation of extracellular matrix components [9]. However, the mechanisms how renal tubulointerstitial fibrosis develops in DN need more clarifications.

Autophagy is a key homeostatic process that is involved in degradation and cycling of protein aggregates, organelles, and other macromolecules [10]. Thus, autophagy is required for the maintenance of cellular homeostasis [11]. Recent evidence has indicated that autophagy participates in the pathogenesis and physiology of types of diseases, including infectious diseases, inflammatory bowel diseases, and neurodegenerative diseases [12]. Additionally, autophagy defects are associated with the pathogenesis of DN [13], and suppression of autophagy aggravates fibrosis in tubular cells [14]. Activation and restoration of autophagy activity may be renoprotective strategies to retard the progression of DN [15]. Although accumulating studies have confirmed the protective role of autophagy preventing the fibrogenesis in DN, the event that initiates this process is largely unknown. Nuclear factor (NF)-κB is thought to be a key regulator in inflammatory response in diabetic kidney disease [16]. Under normal conditions, NF-κB is blocked by its inhibitor, IKB. Persistent activation of NF-κB can promote the release of inflammatory cytokines and enhance the inflammatory response, finally aggravating tissue damage [17–19]. In diabetes, endogenous danger-associated molecular patterns are generated and induce a sterile tubulointerstitial inflammatory response via the NF-κB signaling pathway [16]. Many genes encoding nucleoporins, such as NUP93, NUP107 and NUP205 were reported to participate in the pathogenesis of nephrotic syndrome [20–22]. NUP160 is 1 of up to 60 proteins that constitute the 120-MD nuclear pore complex, which mediates nucleoplasmic transport [23,24]. NUP160 is expressed in both mouse and human cells. It has been reported that NUP160 depletion induces autophagy in mouse podocytes [25].

To data, the regulatory function of NUP160 on autophagy in tubular cells and animal models of DN has not been investigated. In this work, we established DN models in vitro and in vivo to explore the role of NUP160. We hypothesized that NUP160 depletion may have an inhibitory effect on autophagy in DN. Our study may provide a theoretical basic for further investigations of the role of NUP160 in the pathogenesis of DN.

## Material and methods

### Animals

Twenfour healthy male C57BL/6 J mice were purchased from Vital River Co. Ltd. (Beijing, China). All mice were housed in an environment with a 12 h light/dark cycle at 21 ± 3°C. They were allowed to take food and water ad libitum. After one week of acclimation, the mice were divided into the sham group, the DN+adeno-associated virus-negative control (AAV-NC) group, and the DN+AAV-sh-NUP160 group. N = 8 mice each group. To establish a mouse model of DN, 60 mg/kg streptozotocin (STZ; Sigma-Aldrich, USA) in 0.01 M citrate buffer was intraperitoneally injected into the mice for 5 consecutive days [26]. Mice in the sham group were injected with the same amount of citrate buffer. After 48 h, a blood glucose level of ≥ 16.7 mM indicated a successful establishment of DN model. After 2 weeks, AAV-NC or AAV-sh-NUP160 synthesized by HanBio (Shanghai, China) was injected into the mice via the tail vein at 1 × 10^11^ viral genomes each mouse, at a 3-day interval for 8 weeks. All mice were anesthetized with pentobarbital sodium (60 mg/kg). The health of the mice were monitored daily. No mice died accidentally. Body weight and blood glucose of mice were measured twice a week during the experimental period from the third week. In the end, all mice were fasted overnight. Serum and tissues were collected and frozen in liquid nitrogen for biochemical assays and histological analysis. Animal experiments were approved by the Ethics Committee on Animal Experiments of Xinghua People’s Hospital (Jiangsu, China).

## Oral glucose tolerance test (OGTT)

The concentrations of blood glucose were measured using blood samples obtained from the tail vein, with a glucometer (OneTouch Ultra). The oral glucose tolerance test (OGTT) was performed as described [27]. Briefly, for the OGTT, after 8 weeks of intervention, the mice were fasted for 6–8 h. All mice were orally administered 2 g/kg glucose solution. The blood glucose of each group was measured at 0, 30, 60, 90 and 120 min.

## Biochemical measurements in serum

Serum samples were stored at −20°C after separation by centrifugation (3000 g for 15 min at 4°C). Serum insulin, total triglyceride (TG), total cholesterol (TC), low-density lipoprotein cholesterol (LDL-C) and high-density lipoprotein cholesterol (HDL-C), alanine aminotransferase (ALT) and aspartate aminotransferase (AST), urine albumin and urine creatinine, blood urea nitrogen (BUN) and serum creatinine (Scr) were analyzed using the corresponding ELISA kits (Shanghai, China) according to the manufacturer’s instructions. Mouse urinary albumin/urinary creatinine ratio was calculated as mg/mg [28].

## Histological staining

Tissues including kidney and liver were carefully removed, washed with ice-cold saline, and fixed in 10% formalin. All samples were embedded in paraffin and cut into 4-μm-thick slices for morphological and pathological evaluations. Tissue sections were stained with hematoxylin and eosin (H&E), Masson, and periodic acid-silver (PAS) [29], and examined with a light microscope (Olympus, Tokyo, Japan).

## Immunofluorescence assay

Immunofluorescence staining was used to detect the light chain 3 (LC3) level [30]. The tissue sections were incubated with the primary antibody LC3B (ab192890, 1:200, Abcam) for 12 h at 4°C, and subsequently incubated with Fluorescent-conjugated goat anti-rabbit IgG for 2 h at 37°C. DAPI (Sigma-Aldrich) was used to stain the nuclei. Images on the slides were obtained using a confocal fluorescence microscope (Nikon TE300, Japan) and quantified by Image J software (NIH, USA).

## Cell culture

Normal rat kidney tubular epithelial cell line (NRK-52E) and mouse podocyte cell line (MPC5) were purchased from the American Type Culture Collection (Rockville, MD, USA). Cells were cultured in Dulbecco’s modified Eagle’s medium (DMEM) containing 10% fetal bovine serum (Gibco Laboratories, NY, USA), 100 U/ml penicillin, 100 mg/ml streptomycin at 37°C. The incubation was conducted in a Thermo Scientific Heraeus CO_2_ incubator of 5% CO_2_/95% air. All experiments were performed with cells that were passaged 4 to 10 times. For high glucose treatment, cells were serum-starved in medium containing 1% serum for 24 h, followed by treatment with medium containing 5.5 mmol/L glucose (Con), 33 mM high glucose (HG), and 5.5 mM glucose + 27.5 mM mannitol (Mannitol), respectively, for 24 h [31].

## Cell transfection

Short hairpin RNA targeting NUP160 (sh-NUP160) and shRNA-NC (sh-NC) were constructed by Ribobio (GuangDong, China). Cells were seeded in 24-well plates and transfected with 40 nM shRNA vector following the instructions of Lipofectamine 3000 (Invitrogen, USA) as described previously [32].

## Real-time quantitative polymerase chain reaction (RT-qPCR)

Total RNA was extracted from tissues and cells utilizing TRIzol kit (Invitrogen). Next, a Reverse Transcription Kit (Promega, USA) was used for the reverse transcription of total RNA into cDNA. The cDNA was amplified using appropriate primers by Bio-Rad 96fx circulatory (Bio-Rad, USA) with SYBR Green Master Mix (TaKaRa, Japan). The 2^−ΔΔCt^ method was used to calculate NUP160 expression, normalizing to GAPDH [33]. NUP160, forward: 5′-GAGCTTCGTGGAACTGAGC-3′, reverse: 5′-TCCTGGTGATGGAGAACAACT-3′; GAPDH: forward 5ʹ-TCTCTGCTCCTCCCTGTTC-3ʹ, reverse 5ʹ-ACACCGACCTTCACCATCT-3ʹ.

## Western blot

Western blot was performed as previously published [34]. Total protein from tissues and cells was extracted using radioimmunoprecipitation assay lysis buffer (Sangon Biotech, Shanghai, China). Cell or tissue lysates (50 μg protein per lane) were separated on the sodium dodecyl sulfate-polyacrylamide gel electrophoresis (10%) gels for 2 h and then transferred onto the polyvinylidene difluoride membrane (EMD Millipore, USA) for 1 h. After blocking with 5% skimmed milk for 1 h, the membrane was blotted with primary antibodies: NUP160 (PA5-20,565; Thermo Scientific), p62 (ab109012), LC3A/B (ab192890), Collagen IV (Col-IV) (ab236640), interleukin-1β (IL-1β) (ab254360), IL-6 (ab2593341), Tumor necrosis factor alpha (TNF-α) (ab109322), p-NF-κB p65 (ab239882), NF-κB p65 (ab16502), nephrin (ab2163412), podocin (ab181143), α-actinin-4 (ab108201), and GAPDH (ab181602) overnight at 4°C. After washing, the membrane was incubated with secondary antibodies for 1 h at room temperature, and the signals were developed with Immobilon Western Chemiluminescent HRP substrate (EMD Millipore). The images were processed with Image J software (NIH, USA).

## Statistical analysis

Experimental data are shown as the mean ± standard deviation and were analyzed with SPSS20.0 statistics program (IBM, USA). Analysis of data among multiple groups was performed using unpaired t-test and one-way analysis of variance. p < 0.05 was considered statistically significant.

## Results

In this study, we used HG-treated NRK-52E cells and STZ-induced DN mice to explore the function of NUP160 on autophagy and renal fibrosis in vitro and in vivo. We hypothesized that NUP160 depletion may have an inhibitory effect on autophagy. Our results showed that depletion of NUP160 restored autophagy and inhibited inflammation and fibrosis in HG-treated NRK-52E cells and STZ-induced DN mice by downregulating the expression of p62 and Col-Ⅳ, increasing the ratio of LC3II/LC3I, and inactivating NF-κB signaling Moreover, NUP160 knockdown could ameliorate pathological damage in DN mice.

## The NUP160 level is high in HG-treated NRK-52E cells and in DN mice

**Figure 1.**
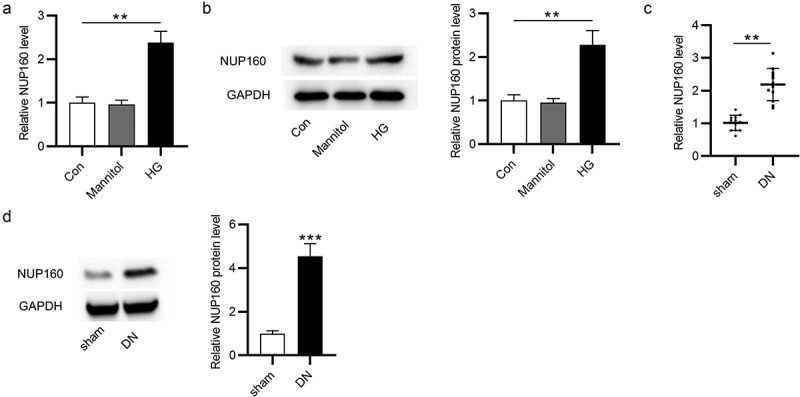
The NUP160 level in HG-treated NRK-52E cells and in DN mice. (a) The NUP160 mRNA level in HG-treated NRK-52E cells was measured by RT-qPCR. (b) The NUP160 protein level in HG-treated NRK-52E cells was determined by western blot. (c) The NUP160 mRNA level in DN mice was measured by RT-qPCR. (d) The NUP160 protein level in DN mice was measured by western blot. **P < 0.01, ***P < 0.001

First, the NUP160 level in NRK-52E cells treated with HG was measured by RT-qPCR and western blot. The results revealed that the expression level of NUP160 mRNA and protein was significantly upregulated in HG-treated NRK-52E cells (Figure 1a and 1b). Moreover, we assessed the level NUP160 in STZ-induced DN mice. It was shown that NUP160 expression was obviously elevated in the kidney tissues of DN mice (Figure 1c and 1d). These results demonstrated the upregulation of NUP160 expression in DN in vitro and in vivo, and thus we hypothesized that NUP160 may participate in the pathology of DN.

## Autophagy is inhibited in DN

**Figure 2.**
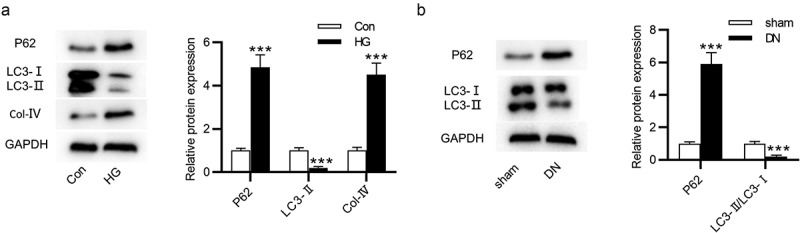
Autophagy level in HG-treated NRK-52E cells and in DN mice. (a) The protein expression of autophagy-related markers and Col-IV in HG-treated NRK-52E cells was assessed by western blot. (b) The protein expression of autophagy-associated markers in DN mice was assessed by western blot. ***P < 0.001

Next, the protein levels of autophagy-associated markers in DN were measured using western blot. The p62 protein expression was increased and the LC3-II/LC3-I ratio was reduced in NRK-52E cells after treatment with HG (Figure 2a). Additionally, the Col-IV level was higher in the HG group than in the control group (Figure 2a). Furthermore, an elevation in p62 expression and a reduction in LC3-II/LC3-I ratio were found in the kidneys of DN mice (Figure 2b).

## NUP160 depletion inhibits autophagy in HG-treated NRK-52E cells

**Figure 3.**
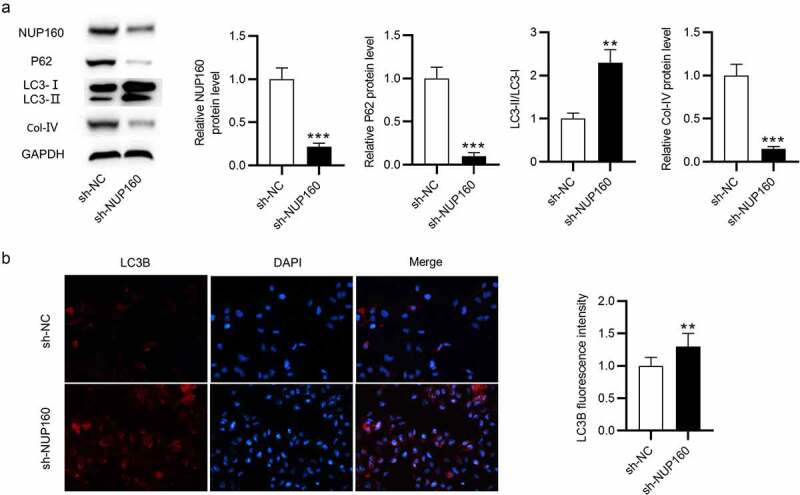
Effects of NUP160 depletion on autophagy in HG-treated NRK-52E cells. (a) The protein levels of NUP160, p62, LC3-I/II, and Col-IV in HG-treated NRK-52E cells transfected with sh-NUP160 were examined by western blot. (b) The expression of LC3B in HG-treated NRK-52E cells transfected with sh-NUP160 was examined using immunofluorescence assay. **P < 0.01, ***P < 0.001

To determine whether NUP160 is related to the regulation of autophagy in NRK-52E cells, we knocked down NUP160 and examined the protein levels of autophagy-associated markers in HG-treated NRK-52E cells after NUP160 silencing. As shown in Figure 3a, the NUP160 protein level was decreased after transfection of sh-NUP160. The p62 protein expression was significantly decreased in HG-treated NRK-52E cells transfected with sh-NUP160 compared to cells transfected with sh-NC. NUP160 depletion also restored the ratio of LC3-II/LC3-I. The Col-IV level was reduced after silencing NUP160. Additionally, the expression of autophagosome adapter protein LC3B was examined using immunofluorescence assay. We observed that the fluorescence intensity of LC3B was notably increased in the sh-NUP160 group compared with the sh-NC group (Figure 3b). Overall, NUP160 depletion promotes autophagy and alleviates fibrosis in HG-treated NRK-52E cells.

## NUP160 depletion suppresses inflammatory response in HG-treated NRK-52E cells

**Figure 4.**
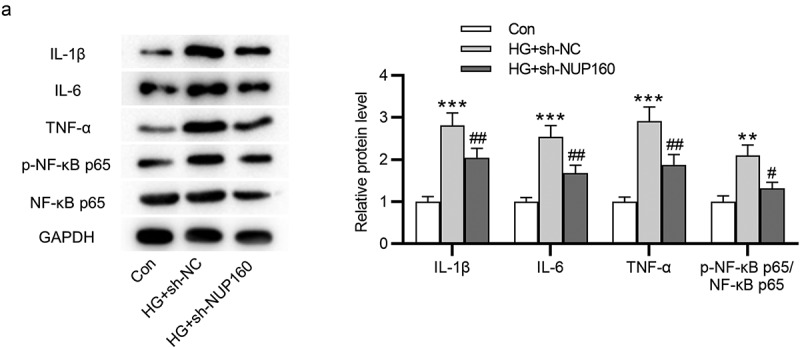
Effects of NUP160 depletion on inflammatory response in HG-treated NRK-52E cells. (a) The protein levels of IL-1β, IL-6, and TNF-α in HG-treated NRK-52E cells transfected with sh-NUP160 were examined by western blot. **P < 0.01, ***P < 0.001 vs. the control group; ^#^P < 0.05, ^##^P < 0.01 vs. the HG+sh-NC group

We further examined the impact of NUP160 in inflammatory response. The results of western blot showed that the protein levels of proinflammatory cytokines IL-1β, IL-6, and TNF-α were increased in HG-treated NRK-52E cells and were then reduced after NUP160 knockdown (Figure 4a). The protein level of NF-κB signaling pathway was detected to explore the mechanisms of NUP160 in inhibition of inflammatory response. We found that the phosphorylation of NF-κB p65 (p-NF-κB p65) in the HG group was higher than that of the control group, while NUP160 knockdown induced a significant decrease in the p-NF-κB p65 level (Figure 4a). Therefore, NUP160 knockdown inhibits inflammatory response by blocking NF-κB signaling pathway.

## NUP160 depletion suppresses podocyte-associated protein disruption

Nephrin, podocin, and α-actinin-4 are important podocyte proteins that help maintain the integrity of the slit diaphragm and prevent proteinuria [35]. We tested whether knockdown of NUP160 could alter the expression of podocyte associated proteins. Here, the mouse glomerular podocyte MPC5 was stimulated with HG, and the sh-NUP160 vector was transfected into MPC5 cells. The results showed that HG could decrease the expression of Nephrin, podocin, and α-actinin-4, while the expression of these proteins were restored after knockdown of NUP160 (Fig. S1A).

## Effects of NUP160 depletion on body weight, blood glucose and insulin in DN mice

**Figure 5.**
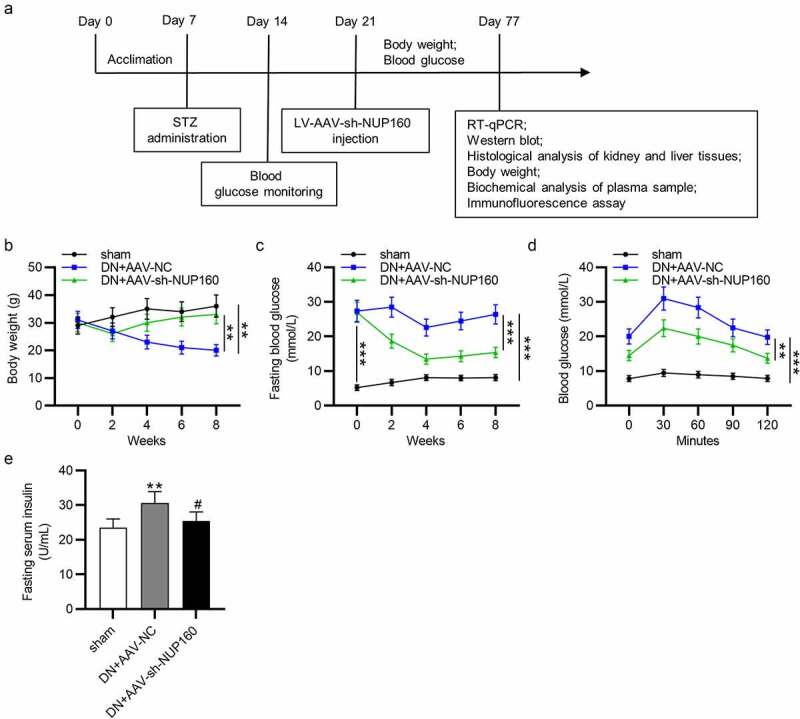
Effects of NUP160 depletion on body weight, blood glucose and insulin in DN mice. (a) The experimental design of the in vivo study is presented in a schematic diagram. (b) Body weight in each group. (c) Fasting blood glucose levels in each group. (d) Changes in the glucose levels in the OGTT. (e) Serum insulin content in each group. **P < 0.01, ***P < 0.001 vs. the sham group; ^#^P < 0.05 vs. the DN+AAV-NC group

To reveal the effects of NUP160 in DN mice, we established a DN model by STZ injection and examined the influence of NUP160 on blood glucose and renal function parameters of the DN mice. The experimental design of the animal study is presented in a schematic diagram (Figure 5a). Compared with the sham group, the body weight in the DN group was significantly decreased, and the body weight was obviously increased after injection of AAV-sh-NUP160 (Figure 5b). At the beginning of the experiment, there were no significant differences in fasting glucose blood levels between the DN+AAV-NC and DN+AAV-sh-NUP160 groups. However, compared with the DN+AAV-NC group, fasting glucose blood levels in the DN+AAV-sh-NUP160 group were significantly reduced at the 8th week (Figure 5c). After oral glucose administration, the blood glucose levels reached their peak value at 30 min in all groups, and then the blood glucose levels gradually returned to their original levels within 120 min (Figure 5d). As shown in Figure 5e, serum insulin levels were markedly higher in the DN+AAV-NC group than the sham group. However, the DN+AAV-sh-NUP160 group exhibited significantly lowered serum insulin levels than the DN+AAV-NC group. The DN mice showed significant hyperlipidemia, high blood urea nitrogen, and creatinine, as shown in Table 1. NUP160 knockdown improved STZ-induced hyperlipidemia by decreasing triacylglycerol and total cholesterol levels. NUP160 knockdown also had an inhibitory effect on the blood urea nitrogen and creatinine. Additionally, the ratio of urine albumin/urine creatinine, an indicator of renal dysfunction, was increased in the DN group and could be alleviated by NUP160 knockdown.

**Table 1.** Biochemical characteristics after 8 weeks of treatment with AAV-sh-NUP160 in DN mice

	Sham	DN+AAV-NC	DN+AAV-sh-NUP160
BUN (mg/dL)	16.39 ± 0.65	30.08 ± 0.97*	25.11 ± 0.62^#^
Cre (mg/dL)	0.24 ± 0.05	0.41 ± 0.03*	0.28 ± 0.03^#^
HDL/LDL	7.05 ± 0.57	7.52 ± 0.70*	7.11 ± 0.61^#^
TG (mg/dL)	56.78 ± 6.94	73.71 ± 5.03*	65.99 ± 3.88^#^
TC (mg/dL)	83.43 ± 4.19	121.26 ± 6.17*	98.85 ± 5.94^#^
UACR (μg/mg)	32.9 ± 3.7	100.8 ± 6.4a*	67.7 ± 2.4^#^

Data are presented as the mean ± SD; n = 8 mice per group. *p < 0.05 indicates significant difference compared with the sham group determined by Student’s t test. ^#^p < 0.05 indicates significant difference compared with the DN+AAV-NC group determined by ANOVA.

BUN: blood urine nitrogen; Cre: creatinine; HDL: high-density lipoprotein; LDL: low-density lipoprotein; TG: triacylglycerol; TC: total cholesterol; UACR: urinary albumin/urinary creatinine.

## Suppression of NUP160 ameliorates kidney and liver injury in DN mice

**Figure 6.**
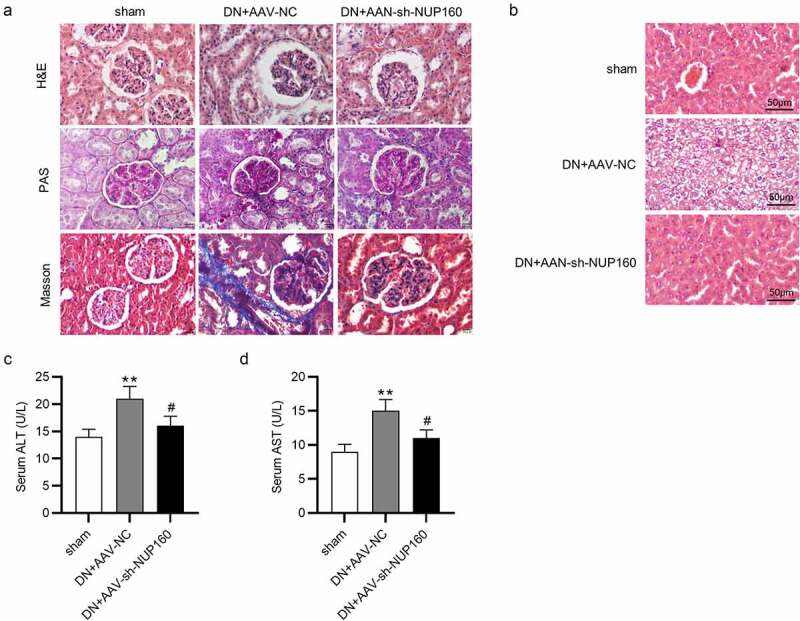
Effects of NUP160 depletion on kidney and liver histopathological changes. (a) Histopathological changes in the kidney. (b) Histopathological changes in the liver. (c) Serum ALT content. (d) Serum AST content. **P < 0.01 vs. the sham group; ^#^P < 0.05 vs. the DN+AAV-NC group

The effects of NUP160 on renal histopathological changes were showed in Figure 6a. The DN group exhibited an increase in mesangial matrix expansion and glomerular volume, basement membrane thickening and significant renal fibrosis accumulation. Pathological injury of the kidney tissues was significantly improved in the DN+AAV-sh-NUP160 group. Furthermore, histological analysis of liver tissues were performed (Figure 6b). In the sham group, the liver lobules were clear, the liver tissue structure was normal, and the shape was intact. However, the hepatocytes in the DN group were largely necrotic, and the cytoplasm was filled with round lipid droplets of different sizes. In the DN+AAV-sh-NUP160 group, the hyperglycemia-induced damage was attenuated. The number of vacuolations was significantly reduced, and the morphology of liver cells was relatively normal. ALT and AST levels can directly represent the degree of liver damage. As shown in Figure 6c and 6d, serum ALT and AST levels were significantly lower in the DN+AAV-sh-NUP160 group than in the DN+AAV-NC group.

## Suppression of NUP160 restores autophagy and inhibits inflammatory response in DN mice

**Figure 7.**
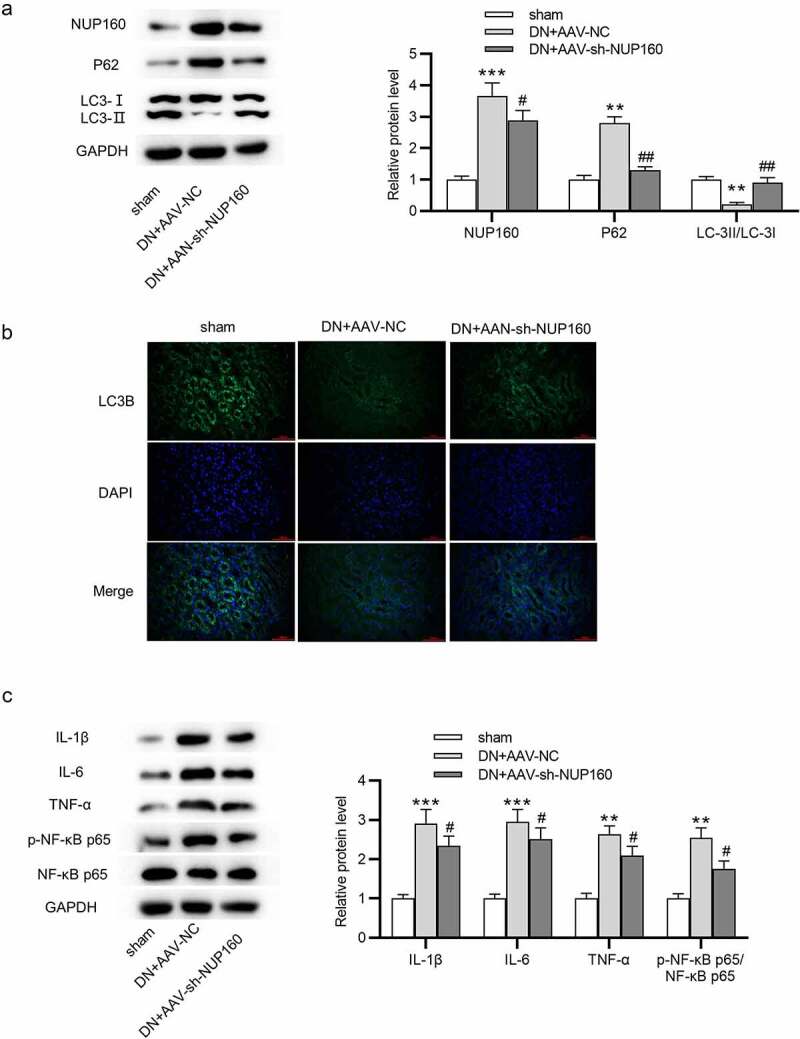
Effects of NUP160 depletion on autophagy and inflammatory response in DN mice. (a) The protein expression of autophagy-related markers and Col-IV in DN mice was determined by western blot. (b) The expression of LC3B in DN mice was determined by immunofluorescence assay. (c) The protein levels of IL-1β, IL-6, and TNF-α in DN mice were examined by western blot. **P < 0.01, ***P < 0.001 vs. the sham group; ^#^P < 0.05, ^##^P < 0.01 vs. the DN+AAV-NC group

We further detected whether NUP160 affects autophagy in vivo. As shown in Figure 7a, the DN+AAV-NC group showed an upregulated expression of NUP160, an increased expression of p62, and a reduced ratio of LC3-II/LC3-I compared with the sham group. The DN+AAV-sh-NUP160 group showed a downregulation expression of NUP160, a decreased expression of p62, and an increased ratio of LC3-II/LC3-I compared to the DN+AAV-NC group, suggesting that silencing NUP160 could promote autophagy in DN mice. Subsequently, we found that the DN+AAV-sh-NUP160 had an elevated LC3B fluorescence intensity compared with the DN+AAV-NC group (Figure 7b). Additionally, NUP160 knockdown reduced the protein levels of proinflammatory cytokines IL-1β, IL-6, and TNF-α in the kidney tissues of DN mice (Figure 7c).

## Discussion

Our findings showed that autophagy was a key factor affecting renal tubulointerstitial fibrosis in DN. Evidence indicates that inactivation of autophagy is a culprit of the pathogenesis of DN, which aggravates fibrosis in tubular cells, and restoration of autophagy activity was found to attenuate cell damage induced by high glucose [36–38]. Therefore, induction of autophagy may be a renoprotective strategy against the progression of DN. Recently, emerging studies have revealed the relationship between tubular autophagy and tubulointerstitial fibrosis [39,40]. For example, Notch homolog 1 (Notch‑1) aggravates renal tubulointerstitial fibrosis in DN by regulating phosphatase and tensin homolog (PTEN) expression via inhibiting autophagy [41]. Silencing microRNA-22 attenuates inhibition of autophagy and Col-IV in HG-treated NRK-52E cells [42]. Dihydromyricetin relieves renal tubulointerstitial fibrosis by activating autophagy in DN [43]. In the current study, western blot results revealed upregulation of p62 and downregulation of LC3 in high glucose-treated NRK-52E cells and in DN mice. Additionally, the levels of Col-IV were increased. Severe renal tubulointerstitial fibrosis was found in DN mice. These findings suggested that autophagy has a key involvement in renal tubulointerstitial fibrosis in DN.

Mutations in the NUP160 gene, which encodes a protein component of the nucleopore complex nucleopore 160 KD, are responsible for inducing steroid-resistant nephrotic syndrome [44]. We found that NUP160 expression was high in HG-treated NRK-52 cells and in STZ-induced DN mice. The role of NUP160 in DN has not been investigated. To identify the relationship between NUP160 and autophagy in DN, the expression of NUP160 was knocked down in HG-treated NRK-52E cells. Our data showed that inhibition of NUP160 could downregulate p62 and upregulate LC3, suggesting that inhibition of NUP160 promotes autophagy in HG-treated NRK-52E cells. Moreover, NUP160 knockdown significantly relieved renal tubulointerstitial fibrosis in DN mice, and restored autophagy in vivo. These findings were consistent with the previous studies demonstrating that NUP160 depletion induces autophagy in mouse podocytes cultured in vitro [25]. Additionally, we also demonstrated that NUP160 knockdown suppressed inflammatory response in vitro and in vivo by reducing the protein levels of proinflammatory cytokines IL-1β, IL-6, and TNF-α. This was the first to reveal the role of NUP160 in regulating inflammatory response. However, some limitations in this study are worth mentioning. First, it would be better to expand the sample size in subsequent experiments to enhance persuasion of our findings. Second, we would also conduct a further research on some potential signaling pathways related to NUP160 in the future study.

## Conclusion

In conclusion, we found that NUP160 depletion could restore autophagy and inhibit fibrosis and inflammatory response in high glucose-treated NRK-52E cells and STZ-induced DN mice. Inhibition of NUP160 might be a promising strategy in preventing renal injury. This study may provide new ideas and bringing new hope for the treatment of DN.

## Supplementary Material

Supplemental Material
