# Supplementary figures and images for: Nucleoporin 160 (NUP160) inhibition alleviates diabetic nephropathy by activating autophagy
Source: Bioengineered. 2021 Sep 17;12(1):6390–402. doi: 10.1080/21655979.2021.1968777 (PMC8806760; doi:10.1080/21655979.2021.1968777)

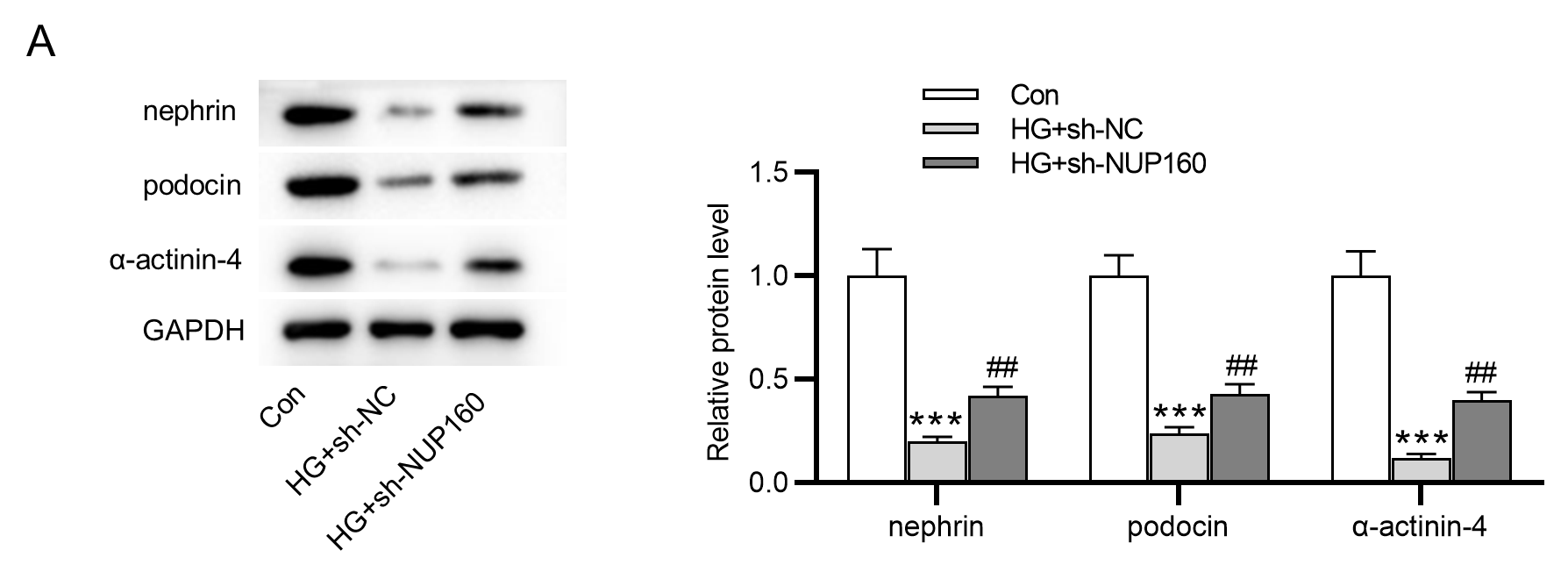

Supplement: Supplemental Material [file KBIE_A_1968777_SM5180.tif]
